# A Comprehensive Dependability Model for QoM-Aware Industrial WSN When Performing Visual Area Coverage in Occluded Scenarios

**DOI:** 10.3390/s20226542

**Published:** 2020-11-16

**Authors:** Thiago C. Jesus, Paulo Portugal, Daniel G. Costa, Francisco Vasques

**Affiliations:** 1 INEGI/INESC-TEC—Faculty of Engineering, University of Porto (FEUP), 4200-465 Porto, Portugal; pportugal@fe.up.pt (P.P.); vasques@fe.up.pt (F.V.); 2 Departmnet of Technology, State University of Feira de Santana (DTEC/UEFS), Feira de Santana 44036-900, Brazil; danielgcosta@uefs.br

**Keywords:** dependability, mathematical modelling, availability, wireless sensor networks, visual sensing, coverage failures

## Abstract

In critical industrial monitoring and control applications, dependability evaluation will be usually required. For wireless sensor networks deployed in industrial plants, dependability evaluation can provide valuable information, enabling proper preventive or contingency measures to assure their correct and safe operation. However, when employing sensor nodes equipped with cameras, visual coverage failures may have a deep impact on the perceived quality of industrial applications, besides the already expected impacts of hardware and connectivity failures. This article proposes a comprehensive mathematical model for dependability evaluation centered on the concept of Quality of Monitoring (QoM), processing availability, reliability and effective coverage parameters in a combined way. Practical evaluation issues are discussed and simulation results are presented to demonstrate how the proposed model can be applied in wireless industrial sensor networks when assessing and enhancing their dependability.

## 1. Introduction

Industrial applications have become more critical and complex over the past years, mainly due the intensification of processes automation and their distributed operation [1]. Many of those applications comprise one or more Wireless Visual Sensor Networks (WVSN), aiming at multi-sensory monitoring for tasks as quality inspection, surveillance and system guidance. Whatever is the nature of such sensors-based networks, their failures can be dangerous to people, lead to environmental damages or even result in economic losses, pushing WVSN applications to pursue a sufficient dependability level [2].

In general, the concept of dependability concerning scalar Wireless Sensor Networks (WSN) has addressed hardware and connectivity failures when modelling faulty conditions [3]. However, for a visual monitoring context, a new type of failure must be also considered, since the way cameras sense the environment is subject to a whole new group of errors and impairments [4]. In fact, visual coverage failures are resulted from total or partial lack of visual information retrieved from cameras, with different particularities depending on the way the environment is perceived. Therefore, when combining hardware, connectivity and coverage failures, more comprehensive dependability evaluations can be performed, potentially benefiting the management of critical sensing applications.

When assessing the dependability of wireless visual sensor networks in industrial environments, in a broader perspective, the concept of Quality of Monitoring (QoM) can be defined. The QoM refers to the capability of a network to perform the expected monitoring functions over a region of interest [5]. In other words, it is not only considered the amount of retrieved visual information when assessing quality, but also definition and sharpness parameters [6]. In practical means, any application may have a minimum expected level of QoM, changing the perception of coverage failures when evaluating dependability: a coverage failure will happen when an application fails to fulfill its expected minimum QoM level.

Several aspects can affect the normal operation of cameras. Common examples are dynamic external (outdoor) factors such as (high/low) luminosity, fog, rain, snow and dust. Particularly for indoor industrial areas, however, there will be two main causes of visual failures that will affect the retrieved visual data and, consequently, the achieved dependability of an application: (1) occlusion and (2) distance of view. Occlusion will be produced by some static or mobile obstacle, potentially reducing a camera’s Field of View (FoV) and indirectly impacting the overall monitoring quality. In a different way, the distance of view is associated to the sharpness of the retrieved visual data [7]. Both types of impairments may simultaneously affect visual sensor nodes and thus they will be modeled as the main causes for QoM degradation.

This article proposes a methodology to allow dependability evaluations of wireless visual sensor networks under the effect of different types of failures. For that, we integrate some previous contributions into a single unified mathematical model, incorporating new elements to better encompass the challenges of industrial monitoring applications. Actually, the proposed methodology comprises common hardware and networking failures associated to scalar sensors, as well as visual coverage failures generated by occlusion or low monitoring quality (modelled as the distance of view). Doing so, researchers acquire a unified model that comprehensively cover frequent but complex failure conditions in such scenarios. Additionally, extensive discussions about practical definitions of the model’s parameters are conducted in this article, addressing an important researching gap that has not been properly considered by the literature. For this purpose, an example of a paper mill application is considered for simulations (Iggesund Paperboard [8]), taking as reference the WirelessHART protocol to connect the sensors [9].

In fact, we have addressed some dependability issues for scalar and visual wireless sensor networks in previous works. In Reference [10], a formal methodology was proposed to model some aspects of hardware and networking failures, focused on scalar sensors. Then, in References [10,11], visual coverage failures were mathematically modelled and processed for dependability assessment, although no dependability evaluation was performed due to the lack of a proper method for this task. In this article, however, we leveraged the mathematical formulations in those work to achieve a more complete model based on different coverage failures that are considered at once. We provide adaptations in the methodology proposed in Reference [10] in order to incorporate this more complete model and consequently to evaluate WVSN in a more comprehensive way. The resulted model and the performed simulations are important contributions that enhance the knowledge in this field, significantly supporting new developments in industrial WSN.

Therefore, the expected contributions of this article can be summarized as follows:

- Integrate different methodologies proposed in previous works, defining a comprehensive unified mathematical model to assess the dependability of wireless visual sensor networks, specially considering the particularities of sensors-based industrial applications;
- Create a consistent reference for quality assessment in critical applications, associating the most relevant failure causes in visual monitoring, which has not been considered before by the literature;
- Discuss how the parameters of the proposed mathematical model can be achieved in practical applications, potentially supporting more effective dependability evaluations;
- Demonstrate a practical use of the proposed methodology considering realistic parameters, which can be easily replicated when evaluating other applications in industrial scenarios.

The remainder of the article is organized as follows. Section 2 presents related works regarding dependability evaluation and coverage failures in wireless visual sensor networks. Fundamental concepts for this work are presented in Section 3. The proposed methodology is described in Section 4, detailing the different evaluation phases. Section 5 presents and discusses important results considering a study case in an industrial scenario. Finally, the conclusions are stated in Section 6.

## 2. Related Works

This article addresses the effects of hardware, connectivity and visual coverage failures on dependability assessment in wireless visual sensor networks. Actually, visual coverage failures are highly impacting on this task, although poorly considered in previous works, specially when performing monitoring tasks associated to the coverage of a defined area. In fact, visual coverage failures are related to insufficient area coverage, which may be resulted from different causes such as incorrect deployment, occlusion and low monitoring quality due to visual effects. Nevertheless, hardware and connectivity (networking) failures may also significantly impact the systems’ dependability, demanding proper considering. This section presents some related works in those research areas, pointing out important references and development directions for the proposed model.

### 2.1. Occlusion and QoM when Considering Area Coverage

These are some important topics that have been considered in different ways when performing visual monitoring or when assessing monitoring quality.

References [12,13] addressed full-view area coverage in WVSN, aimed at the computing of the total area that is being covered by the sensors. For that, those works take into account the orientation of each point or region that is faced by the sensors. In these cases, the Quality of Monitoring is directly related with the facing angles.

The authors of References [14] also addressed area coverage, focused in the automatic determination of the number, position and pan-tilt-zoom setting of a set of cameras to maximize the coverage. In this process, the authors consider QoM by handling the distortion models that can affect the quality and usability of the acquired data.

For Shriwastav and Song [15], area coverage is considered in a different class of WVSN, the Unmanned Aerial Vehicle (UAV) networks. In this case, each UAV is equipped with a camera, being responsible to perform a persistent full coverage of the area. If eventually an UAV fails, it is considered “unrecoverable” and the network must be redeployed, adjusting position and loitering movements to keep the area covered. An important aspect is that the authors consider the loitering altitude constant in order to keep the coverage quality constant.

Although they consider QoM in area coverage, neither the works in References [12,13,14,15] actually compute a sensor node’s or network’s QoM. The work in Tao et al. [6] overcomes this issue by defining a weighted sensing quality, which is calculated over a predefined deployment scheme. In that case, it is discussed the importance of sensing area to establish QoM in a full area coverage scenario. However, several restrictions are imposed to the network regarding to the nodes’ position, orientation and viewing angle.

In Reference [5], QoM is addressed extrapolating several deployment constraints imposed in Reference [6], such as cameras’ position, orientation and viewing angle. New QoM-based metrics are proposed, allowing new researches related to optimization, comparison or exploitation of different quality aspects, such as redundancy and dependability.

Some papers also relate QoM with occlusion, which is commonly addressed as an aspect that reduces the coverage area. In Reference [16], authors do not address the overall area coverage quality, since they consider occlusion to determine a satisfactory sensing quality of a monitoring area, when a set of waypoints are chosen for taking picture of some objects. The proposed coverage technique is used to achieve energy efficiency by minimizing the maximum energy consumption among sensor nodes.

Still considering this overall scenario, Costa et al. [17] and Jesus et al. [11] address occlusion in a more significant way, proposing computation of area coverage under occlusion. The work in Costa et al. [17] proposed a model to compute occlusion caused by simplified static obstacles, which were modeled as 2D walls (lines). In that work, occlusion affected FoV overlapping and selection of redundant nodes, directly impacting on availability assessment. Authors in Jesus et al. [11] provide an enhanced occlusion approach, modelling the obstacles as mobile 2D rectangles, which are a better and reasonable approximation of real objects. The goal of that work was the selection of visual nodes based on effective viewed area to better perform existing optimization or quality assessment approaches.

### 2.2. Dependability Assessment

Quality of Monitoring and occlusion can negatively affect a system’s dependability, being important the using of a method to assess their impact. In case of distributed sensor-systems (Cyber-Physical Systems, Internet of Things (IoT), WSN), dependability assessment has been more seriously addressed in recent years, generally considering aspects involving hardware components of sensor/actuator nodes, network communication elements and network lifetime.

The work in Andrade and Nogueira [18] proposes a dependability evaluation approach based on stochastic Petri nets (SPN). That approach analyses disaster recovery solutions for IoT infrastructures, evaluating system reliability and availability, considering energy consumption and network communication.

In Reference [19], a methodology for automatic generation of analytical dependability models based on fault trees is proposed for industrial applications. That methodology is integrated with the SHARPE tool and evaluates the behavior of a scalar wireless sensor networks with regard to some attributes, such as reliability and availability. That methodology was later improved in References [10,20].

The authors of Reference [20] perform availability assessments in wireless visual sensor networks. That evaluation is associated to target monitoring, considering hardware, communication and coverage failures, which were modeled as “loss of view” over targets. However, the proposal presents some limitations, such as not considering the effects of routing protocols or ignoring different importance degrees of sensor nodes on network failure conditions generation.

The aforementioned limitations are circumvented in Reference [10], which considered different routing strategies in the context of area coverage monitoring. That way, it is possible to specify network failure conditions as a function of a coverage objective. However, none of these methodologies considered the effect of visual coverage failures by occlusion and loss of Quality of Monitoring.

Actually, although several papers have addressed issues such as visual occlusion, Quality of Monitoring and dependability assessment on WVSN, such issues have been considered separately. In a different way, this article integrates all those issues in a unified dependability evaluation model that has not been proposed before, to the best of our knowledge.

Table 1 summarizes the discussed related works in areas related to QoM, occlusion, WSN dependability evaluation and modelling of visual coverage failures.

## 3. Definitions and Basic Concepts

### 3.1. Area Coverage

**Figure 1:**
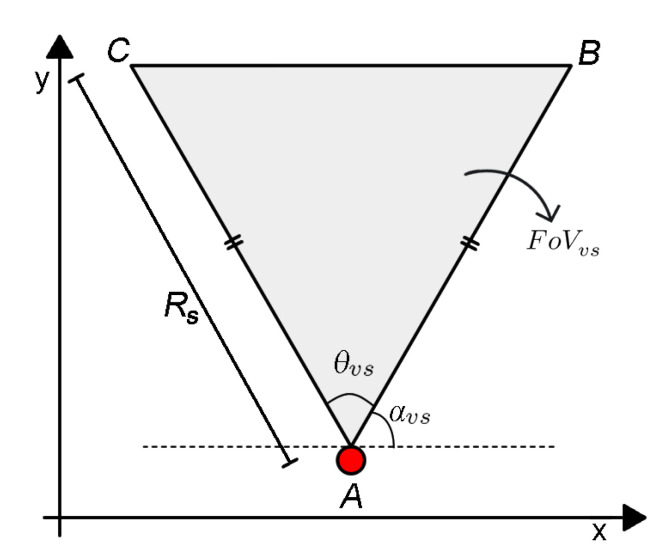
Field of View of a visual sensor.

The WVSN considered in this article is composed of a set of visual sensor nodes $VS=\left\{vs_{1},vs_{2},\;...,\;vs_{n}\right\}$, which are deployed over a two-dimensional area A. Each camera may assume different values for their parameters, however, without loss of generality, we consider in this work that all visual sensors are identical and so they are configured with the same values [21]. Each sensor is expected to be equipped with a camera, having a viewing angle $\theta_{vs}$ and an orientation $\alpha_{vs}$, as shown in Figure 1, where the camera is represented by the small red circle. Each camera also presents a sensing radius $R_{s}$ as the approximation of the distance between the nearest and farthest point that can be sharply sensed.

The field of view of any visual sensor is defined as the area of an isosceles triangle composed of three vertices, *A*, *B* and *C* (see Figure 1), being $\left(A_{x},A_{y}\right)$ the Cartesian coordinates of the sensor node. The coordinates of vertices *B* and *C* can be computed according to Equation (1) and the FoV of any visual sensor vs is the area of the triangle △ABC, which can be computed using trigonometry, as expressed in Equation (2) [20].

(1) $$\begin{array}{c} B_{x}=A_{x}+R_{s}\cos\left(\alpha_{vs}\right)\\ B_{y}=A_{y}+R_{s}\sin\left(\alpha_{vs}\right)\\ C_{x}=A_{x}+R_{s}\cos\left(\left(\alpha_{vs}+\theta_{vs}\right)\;\mathrm{mod}\;2\pi \right)\\ C_{y}=A_{y}+R_{s}\sin\left(\left(\alpha_{vs}+\theta_{vs}\right)\;\mathrm{mod}\;2\pi \right) \end{array}$$

(2) $$FoV_{vs}=\frac{R_{s}^{2}\cdot \sin\left(\theta_{vs}\right)}{2}.$$

**Figure 2:**
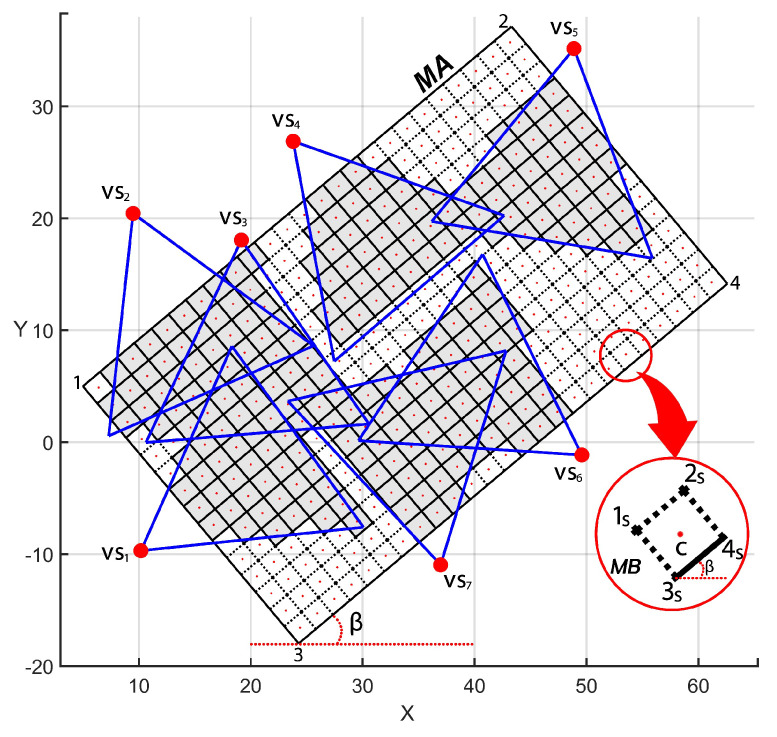
Monitoring area being covered by visual sensor nodes $vs_{i}$, i=1,⋯,7 [5].

The monitoring field A may be composed of one or more *Monitoring Areas* (MA), each one described as a rectangle defined by its origins (x1, y1), a width *w*, a height *h* and a rotation angle β, as shown in Figure 2. The coordinates of the other vertices of a MA (vertices 2, 3 and 4) can be computed as presented in Equation (3). A MA is the area of interest of a visual application and only visual information from this sub-region is relevant to the considered WVSN.

(3) $$\begin{array}{c} x2=x1+w.\cos(\beta ),\;\;\;\;\;\;\;\;\;\;\;\;\;\;\;\;\;\;\;\;\;y2=y1+w.\sin(\beta )\\ x3=x1+h.\sin(\beta ),\;\;\;\;\;\;\;\;\;\;\;\;\;\;\;\;\;\;\;\;\;\;y3=y1-h.\cos(\beta )\\ x4=x1+h.\sin(\beta )+w.\cos(\beta ),\;\;\;y4=y1-h.\cos(\beta )+w.\sin(\beta ). \end{array}$$

A MA can be divided into smaller regions, called *Monitoring Blocks* (MB), each one defined as a rectangle represented by its origins (x1s, y1s), a width ws, a height hs, a centroid (xc, yc) and the same rotation angle β of the MA, as shown in Figure 2. The coordinates of the other vertices of a MB (vertices 2s, 3s and 4s) can be calculated similarly to Equation (3). Thus, a MA is composed of a grid of MB with size *M* × *N*. In this case, the “area coverage” problem can be indirectly approached as several “target coverage” problems, where each point (xc, yc) is a target with infinitesimal size. This is an important abstraction aimed at higher efficiency, while keeping the computational cost low, as it was discussed in Reference [22]. In that paper, the monitoring blocks approach for area calculation has been proven to be a good approximation in terms of accuracy, with low computational cost.

A single monitoring block mb is considered to be covered by a visual sensor node vs if its center (xc,yc) is within the polygon area of $FoV_{vs}$. In this configuration, the area of a mb (ws×hs) is accounted to the total coverage area [22]. A covered MB is represented by notation $mb\in FoV_{vs}$. This definition can be extended for a set of visual sensors VS according to Equation (4). The total area covered by VS is computed according to Equation (5), where $mb_{j,l}$ is a MB at the position (j,l) of the grid.

(4) $$cover\left(mb,VS\right)=\left\{\begin{array}{cc} 1, & \mathrm{if}\;\;\exists vs\in VS|mb\in FoV_{vs}\\ 0, & \mathrm{otherwise} \end{array}\right.$$

(5) $$CA_{mb}\left(VS\right)=ws\cdot hs\cdot \sum_{j=1}^{M}\sum_{l=1}^{N}cover\left(mb_{j,l},VS\right).$$

### 3.2. Quality of Monitoring

Quality of Monitoring is a generic term used to refer to the congruence between the expected and performed monitoring functions over a region of interest. In the case of a WVSN, QoM can be related to how good are the visual data definition and sharpness provided by a set of cameras for a region located on a certain distance dov (distance of view). The farther is the monitored region from the camera, the lower is the level of details related to that region in captured images, and consequently, the lower should be the amount of visual information extracted from that region. The QoM modeling used as reference in this article was initially proposed in Reference [5], being summarized next.

**Figure 3:**
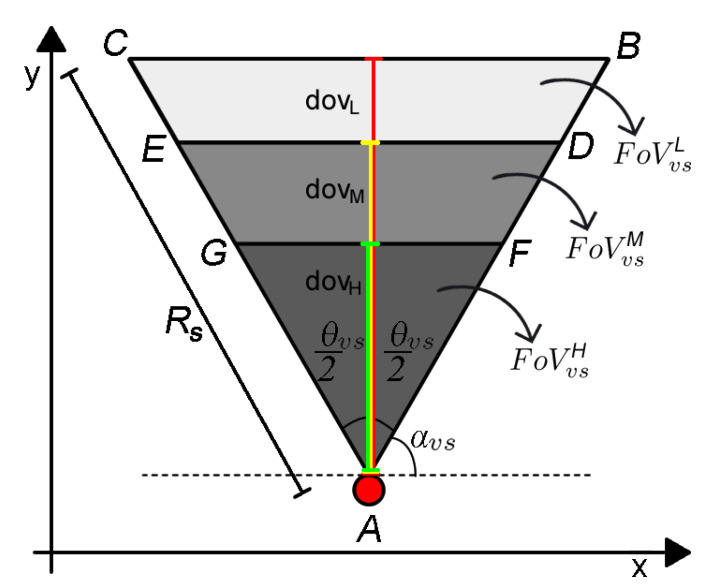
Quality model of visual sensor’s Field of View [5].

A sensor node vs∈VS has its FoV divided into several disjoint sub-regions. In this article it is considered only three sub-regions ($FoV_{vs}^{H}$, $FoV_{vs}^{M}$ and $FoV_{vs}^{L}$), which determine the visual levels with *high*, *medium* and *low* quality, respectively. The first level is defined by an isosceles triangle △AFG with its high $dov_{H}$ defined as the distance from vertex *A* to the end of $FoV_{vs}^{H}$. The second and third levels are defined by isosceles trapezoids ⬠DEGF and ⬠BCED with highs equal to ($dov_{M}-dov_{H}$) and ($dov_{L}-dov_{M}$), respectively, as depicted in Figure 3. It is important to notice that the sensor FoV is not modified. It is only re-interpreted, being $FoV_{vs}=FoV_{vs}^{H}\cup FoV_{vs}^{M}\cup FoV_{vs}^{L}$ and $FoV_{vs}^{H}\cap FoV_{vs}^{M}\cap FoV_{vs}^{L}=\emptyset$.

The distances $dov_{H}$, $dov_{M}$ and $dov_{L}$ can be computed according to Equation (6) and the proportion of $dov_{H}$ and $dov_{M}$ with respect to $dov_{L}$ can be defined freely, considering the application requirements and camera’s constraints. The coordinates of vertices *D*, *E*, *F* and *G* can be computed similarly to Equation (1).

(6) dovL=Rs.cosθvsθvs22dovM=33/44dovLdovH=22/44dovL.

The QoM can be assessed by the Area Quality Metric (AQM), which is computed considering that each monitoring block $mb\in FoV_{vs}$ receives a weight $w_{l}$ which is the weight of the FoV sub-region of vs where mb can be viewed, as expressed in Equation (7). The weight values for each visual level are assigned as $w_{1}=1$ for $FoV_{vs}^{H}$, $w_{2}=0.5$ for $FoV_{vs}^{M}$ and $w_{3}=0.25$ for $FoV_{vs}^{L}$.

(7) $$w_{l}\left(mb,vs\right)=\left\{\begin{array}{cc} w_{1}, & \mathrm{if}\;\;mb\in FoV_{vs}^{H}\\ w_{2}, & \mathrm{if}\;\;mb\in FoV_{vs}^{M}\\ w_{3}, & \mathrm{if}\;\;mb\in FoV_{vs}^{L} \end{array}\right.$$

If a monitoring block mb is redundantly covered by a visual sensor set VS, then the weight of mb is the maximum weight among the associated sensor, as expressed in Equation (8).

(8) $$w_{\max}\left(mb,VS\right)=\max\left({\left.wl\left(mb,vs\right)\right|}_{\forall vs\in VS}\right).$$

**Figure 4:**
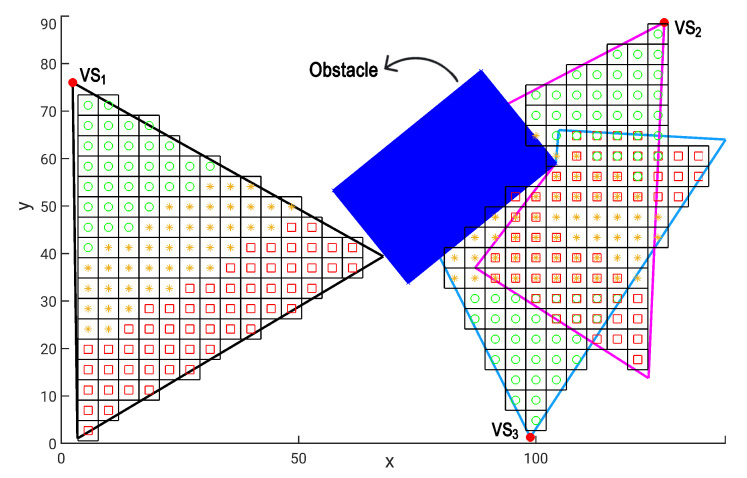
Quality of the performed area coverage when viewing monitoring blocks.

Figure 4 illustrates the mapping of monitoring quality of the MB covered by three visual sensors, including the overlapping considerations. The visual sensor $vs_{1}$ performs a normal monitoring, while the sensors $vs_{2}$ ans $vs_{3}$ have their FoV occluded by an obstacle. The occlusion scenario is addressed in Section 3.3. The MB marked with a green circle are in *level 1* (highest quality), while the ones marked with a yellow star are in *level 2* (medium quality) and the MB marked with a red square are in *level 3* (lowest quality). Notice that there are some MB marked with more than one symbol: those MB are redundantly monitored by more than one visual sensor and its assigned weight is that one related to the highest quality.

The metric AQM is presented in Equations (9) and (10). AQM provides a global perspective of the quality of monitoring, indicating the percentage of the equivalent monitoring blocks related to the entire monitoring field. $AQM_{abs}$ is an intermediate metric that provides an absolute perspective of the quality of monitoring, indicating the equivalent quantity of monitoring blocks.

(9) $$AQM_{abs}\left(VS\right)=hs\cdot ws\cdot \sum_{j=1}^{M}\sum_{l=1}^{N}w_{\max}\left(mb_{j,l},VS\right)$$

(10) $$AQM\left(VS\right)=\frac{AQM_{abs}\left(VS\right)}{h\cdot w}.$$

### 3.3. Occlusion

In this article, we take as reference the modeling of obstacles and the computation of occlusions as proposed in Reference [11]. The obstacles are considered being 2D rectangles, which is a reasonable simplification of real objects, providing a practical and yet effective mathematical model. An obstacle can be defined by its origins ($x1_{obs}$, $y1_{obs}$), a width $w_{obs}$, a height $h_{obs}$ and an orientation angle $\beta_{obs}$. The remaining vertices can be computed similarly to Equation (3).

In order to compute the visual occlusion caused by an obstacle, the field of view of each visual sensor is processed against a set of obstacles, computing *occlusion vertices*, which are mostly resulted from the intersection of the obstacles’ edges with the visual sensors’ edges, resulting in convex or concave polygons for each visual sensor. This visual coverage area derived from an original FoV is defined as the *Occluded FoV* (OFoV). Hence, the computed area of each OFoV jointly with their associated QoM will be exploited to identify if a certain visual sensor is under a coverage failure. More details about how to compute the occlusion vertices and the OFoV can be found in Reference [11].

Figure 4 shows an example of OFoV from sensors $vs_{2}$ ans $vs_{3}$. Obviously, an occlusion reduces the covered area and the quality of monitoring, which can configure a visual coverage failure, directly affecting a system’s dependability.

## 4. Proposed Methodology

The dependability assessment methodology proposed in this article is aimed at solving an important quality evaluation gap in critical applications that employ scalar and visual sensors to perform monitoring tasks. For that, we integrate and unify some previous methodologies [5,10,11], achieving a more complete mathematical solution for such scenarios. In this way, the proposed methodology for dependability evaluation considers different visual coverage failures, which occur when the required area coverage or quality of monitoring level are not fulfilled, either by an incorrect deployment or by occlusion.

**Figure 5:**
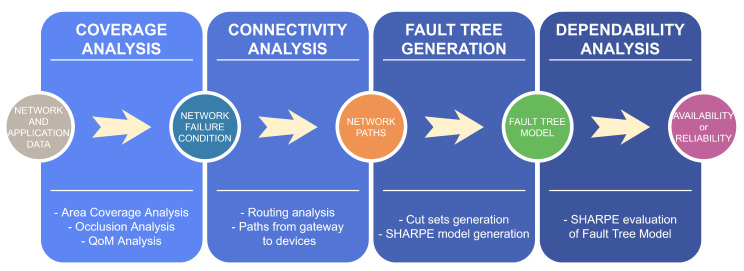
Flowchart of the proposed evaluation methodology.

The flowchart of the proposed evaluation methodology is presented in Figure 5 and depicts the methodology in four stages: *coverage analysis*, *connectivity analysis*, *Fault Tree generation* and *dependability analysis*. Backwards, the *dependability analysis* (the last stage) is performed in terms of its quantitative attributes (reliability and availability), as it is commonly addressed in the literature [23,24,25,26]. For this task, the SHARPE (Symbolic Hierarchical Automated Reliability and Performance Evaluator) tool [27] is used to numerically compute a hierarchical model of a fault tree embedded with Markov chains. It is worthy to remark that it is an automated methodology, which means that, besides the input data at the initial stage of the methodology, no further interaction with the user is required for dependability assessment.

The hierarchical model is built in the previous stage of the methodology, the *Fault Tree generation*. For this, the proposed methodology assumes a network that is organized in a tree topology, rooted at the gateway node. This node manages the system monitoring application, receiving data from the other visual nodes. Therefore, a visual application will fail if the network is not capable to deliver the required visual information to the gateway. This can happen due to failures of network nodes that compose the *Network Failure Condition* (NFC), which is a logical expression that indicates which nodes or combination of nodes ($fc\_Comb_{i}$) must fail to lead to the application failure. The nodes participating in the NFC are called *essential nodes*.

**Figure 6:**
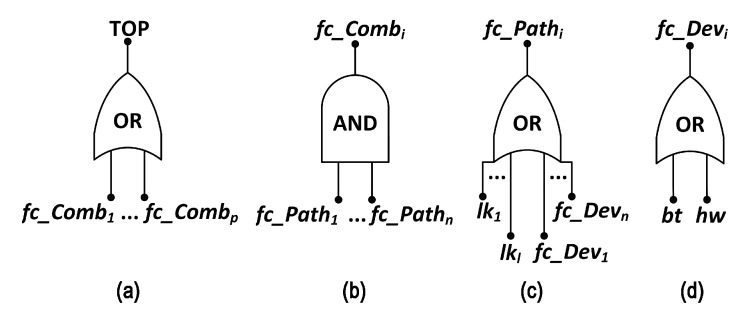
Fault Tree models of (**a**) network, (**b**) combinations, (**c**) paths and (**d**) devices [10].

The NFC is used to build a fault tree. In this case, an application failure is identified whether the NFC is assessed equals to *true*, which is equivalent to the TOP event of the fault tree be assessed equals to *true*. Figure 6 shows the gates of the fault tree structure considered in the methodology.

**Figure 7:**
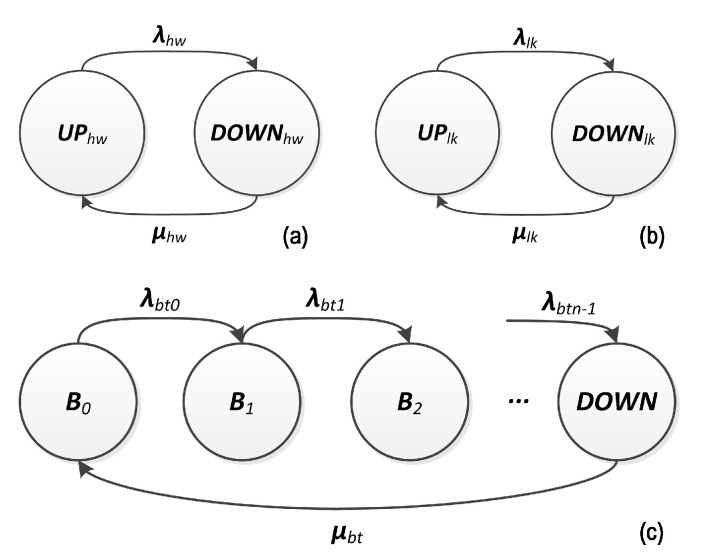
Markov Chain models of (**a**) hardware, (**b**) link and (**c**) battery [10].

A combination of nodes (devices) fails if all paths ($fc\_Path_{i}$) that connect the gateway to each node in the combination fail. A path fails if any link ($lk_{i}$) or node ($fc\_Dev_{i}$) in the path from the node to gateway fails. Finally, a node fails if its hardware (hw) fails or if its battery discharges (bt) [10]. The basic events $lk_{i}$, hw and bt must be assigned to battery, hardware and link dependability functions (reliability or availability functions, according to the evaluation interest), resulting from the respective Markov chain evaluation, according to Figure 7. These Markov chains describe the component behavior through two states: UP (operational) and DOWN (failed). Transitions between these two Markov chain states are described by the failure ($\lambda_{hw}$, $\lambda_{lk}$) and repair rates ($\mu_{hw}$, $\mu_{lk}$, $\mu_{bt}$), respectively. In case of battery, the states $B_{0},B_{1},\cdots ,B_{n-1}$ represent progressive discharging states, the state $B_{n}$ represents a fully discharge battery and the transition rates $\lambda_{btk}$, k=0,⋯,n−1, are the battery discharge rates in function of the power consumption.

At the *connectivity analysis* step (the second stage in Figure 5), the paths from the nodes of NFC to the gateway are represented in the fault tree, and they are defined according to the routing strategy. For this, it is necessary to know the NFC of the considered network.

At this point, we extended our previous work presented in Reference [10]. In that work, the NFC was generated based on the covered area. However, as important as the amount of visual information, the quality of the gathered information is crucial for applications. Actually, both the amount and quality of this information can be jeopardized by occlusion, leading to a dependability decreasing.

That way, these coverage failures in the considered network have to be properly processed. In fact, coverage failures may happen in different ways and in different moments of the network lifetime. Nevertheless, in order to properly evaluate the dependability of the applications, the proposed methodology processes all coverage failures at once, achieving a final dependability perception. Therefore, the standard procedure is to sequentially compute all modelled coverage failures, first computing occlusions on all visual sensors and then processing the QoM associated to the Depth of View based on Occluded FoV (OFoV).

**Figure 8:**
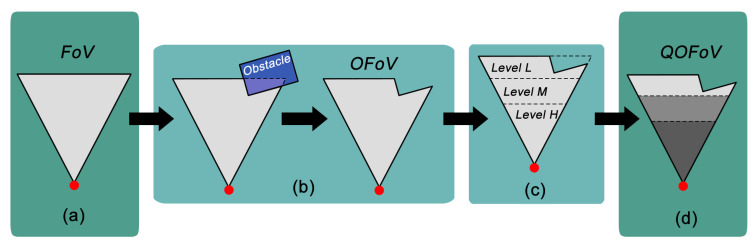
A sensor’s field of view in different processing steps: (**a**) Original coverage, (**b**) Field of View (FoV) under occlusion, (**c**) Quality of Monitoring (QoM)-aware FoV and (**d**) Quality-Occluded FoV (QOFoV) for both occlusion and QoM-aware conditions.

The computation of the OFoV will consider all static or moving obstacles that can produce some level of occlusion on the deployed visual sensors. For that, the formulations in Reference [11] can be leveraged to achieve a set of Occluded FoV for the considered network, reflecting the impacts of the obstacles along the time. Then, the QoM of that set of active visual sensors can be also processed based on the definitions in Reference [5], but now assuming that the FoV of any visual sensors may be not an isosceles triangle. In other words, two sequential layers of coverage failures will be computed for the visual sensors, and their resulted coverage on the monitored field will already reflect the impacts of both types of coverage failures. This general processing principle is depicted in Figure 8.

As an important remark in Figure 8, while a visual sensor may be not occluded by an obstacle, which may result in an OFoV similar to the original FoV, the QoM-aware FoV of that sensor will be always computed.

Based on the processing of these coverage failures, we define the NFC considering that the application will fail if a combination of nodes are not able to cover a minimum amount of covered area, a minimum quality of monitoring and also considering the minimum percentage of FoV of each visual sensor. For this last case, it is considered that, if a single sensor node cannot cover its minimum FoV, its camera is turned off, and the node operates as a *relay node*, that is, a node that only re-transmits messages, therefore consuming less power, saving battery, extending its lifetime, and so increasing the system dependability.

Initially, the methodology requires some supplementary data from the user in order to characterize the network and the application requirements. This information is related to network configuration (e.g., topology), visual coverage attributes, nodes, the evaluation process itself and specially the application requirements, which is the minimum visual coverage percentage ($CA_{min}$), the minimum QoM ($AQM_{min}$) and the minimum FoV ($FoV_{min}$).

The Algorithm 1 describes the redesigned NFC generation, when a combination of sensor nodes is included into the NFC expression if it provides the required area coverage and QoM (Line 33). Doing so, the dependability evaluation can be carried out considering the mathematical parameters of the scenario.

The Algorithm 1 starts computing the OFoV of each visual sensor according to Jesus et al. [11] (Line 2). Then, it is verified which monitoring blocks are covered by each visual sensor and their respective QoM. For this, the procedure inpolygon() in Lines 6, 11–13 checks if the center of a mb is within of the OFoV of a visual sensor vs, and within of one of its quality level sub-regions $FoV_{vs}^{H}$, $FoV_{vs}^{M}$ or $FoV_{vs}^{L}$, respectively. This procedure implements a Ray casting algorithm [28,29]. Notice that we first verify if a mb is within of the OFoV of vs (Line 6). This is important to not consider monitoring blocks in occluded areas. According to how a sensor vs covers a monitoring block mb, the functions $w_{l}\left(mb,vs\right)$ and $cover\left(mb,vs\right)$ are updated (Lines 14–25). This information is used to proper compute the overall coverage area $CA_{mb}\left(VS^{\prime }\right)$ and QoM $AQM\left(VS^{\prime }\right)$ provided by a subset of visual sensor $VS^{\prime }\subseteq VS$ (Lines 30–32). Finally, the NFC is updated if the sensors in this subset are able to fulfill the application requirements together, thatis, if they cover an area greater or equal to $CA_{min}$ with QoM greater or equal to $AQM_{min}$ (Lines 33–34).
**Algorithm 1:** NFC.
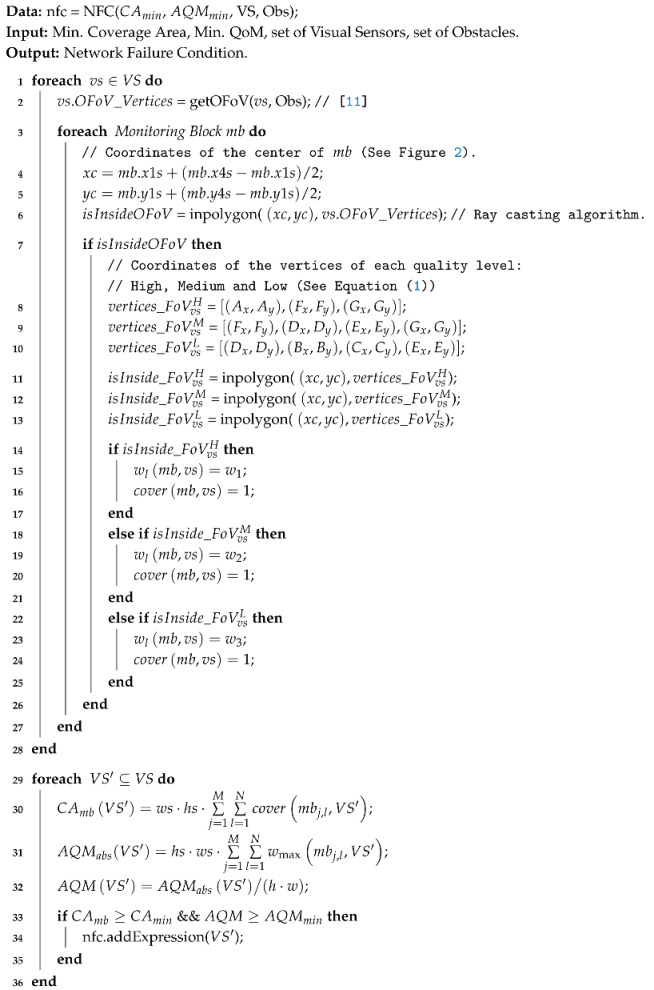


Following all these steps, the dependability of any wireless visual sensor networks can be evaluated, potentially supporting in the designing, deployment and management of those networks. At this point, what is required is the proper setting of the evaluation parameters, which will be discussed in next section.

## 5. Practical Dependability Assessment

In this section it is presented the dependability assessment of an industrial application in order to show the applicability of the proposed methodology. Actually, for this task, the methodology parameters could be empirically set. However, in order to facilitate the usage of the methodology in real scenarios, some approaches are enhanced in relation of previous works, and employed to determine most of these parameters.

There are many possibilities to determine each parameter. Of course, each approach will be as accurate as the considered detailing level. In fact, we are not focused on strictly determining the parameters, but providing possible solutions when defining them. With a realistic parameters setting, we can more confidently evaluate the impact of visual coverage failures on the system dependability.

**Figure 9:**
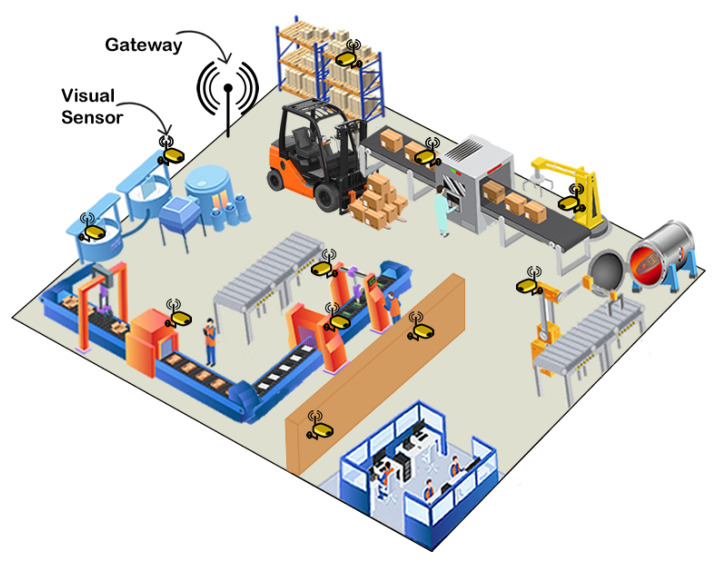
Shop floor layout.

**Figure 10:**
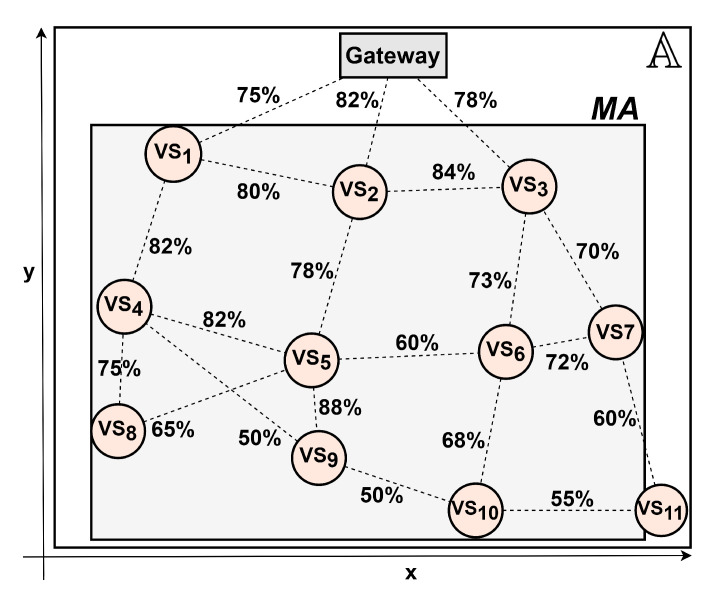
Shop floor network.

The proposed scenario used as case study is a merging of specifications of two real industrial shop floors into a single realistic model. This industrial scenario is similar to a paper mill proposed in Reference [8] (Iggesund Paperboard), which contains machinery such as boilers, mixing and storage tanks, conveyors and storage racks. We assume that the factory occupy an area A of 250 m × 200 m, where the monitored area MA is 240 m × 180 m. Over this MA are deployed 11 visual sensor nodes and one gateway node, according to the industrial wireless network presented in Reference [9], implementing a network topology according to the WirelessHART standard (one of the most adopted wireless communication protocol in industrial applications [30]). The visual nodes are oriented in a way to monitor regions of production more susceptible to failures, accidents and intrusion. Figure 9 shows the shop floor layout and Figure 10 presents the network topology. The percentage on the edges of the graph are the packet reception rates (PRR), which are link quality metrics that will be used to determine the link failure rates.

Next we show how to obtain the dependability methodology parameters from the component specifications. The interested reader is referred to Reference [10] for more details on the parameters meaning.

### 5.1. Experimental Parameter Settings

The methodology requires some data related to visual nodes, components failure and repair rates, network communication (topology, routing, radio range, etc.) and the application requirements. We assume that all the visual sensor nodes are composed of a camera FLIR BLACKFLY${}^{®}$S BFS-U3-13Y3 and that they communicate through an IEEE 802.15.4 radio interface at 2.4 GHz, using a CC2420 antenna which can support WirelessHART setting [31]. Each node is powered by a typical battery of WirelessHART field devices, a lithium battery unit BU 191 [32] and they communicate based on flooding routing strategy.

We assume that the useful visual information from cameras can be retrieved of a distance not higher than 75 m from the camera, which implies in a sensing radius of $R_{s}=75$ m with the viewing angle of $\theta_{vs}=60^{\circ }$ (an intermediate value and also the average viewing angle of several commercial cameras). Regarding the monitored area, its dimensions are w=240 m and h=180 m, divided into monitoring blocks with ws=hs=2.15 m (approximately 9% of the FoV [22]) and β = $0^{\circ }$. The position of each sensor node was generated according to Figure 10. The dependability parameters, according to the information available in References [32,33,34], were pessimistically set, with the hardware failure and repair rates $\lambda_{hw}=2.2831\times 10^{-5}$ and $\mu_{hw}$ = 0.0833, respectively, considering the worst case scenario of the hardware components specifications. In this article, all failures, repair and discharging rates are measured by hour.

The battery has an initial capacity $c_{0}=19$ Ah rated by H=1 h and a Peukert’s constant η=1.1. Considering that the average continuous discharge current needed to supply the sensor node (radio, camera, etc.) is about I=150 mA, then the discharge rates of a 4-discharging stages of the battery model are $\lambda_{bt0}$ = 0.0225, $\lambda_{bt1}$ = 0.0197, $\lambda_{bt2}$ = 0.0187 and $\lambda_{bt3}$ = 0.0181 h. Once discharged, we consider that it takes 2 hours to repair (replace or recharge) the battery, so $\mu_{bt}=0.5$.

The estimation of the link rates is more complex since the link has a transient behavior, which means that a failed link will reestablish its connection after a while, without an external intervention. A link failure could be caused by an obstruction produced by an external object, shadowing, signal attenuation, multi-path fading, radio interference, background noise, an occupied channel or a data collision [35]. Due to several uncertainties or difficulties when modelling these aspects, usually resulted from a lack of sufficient data, we consider the fuzzy model approach presented in Reference [36] to estimate link failure rates based on link quality metrics. These metrics could be, for instance, packet reception rate, packet error rate (PER), link failure probability or time to link failure (TLF). They can be obtained by simulation [37], estimation [38] or by radio propagation models [39].

The approach proposed in Reference [36] expresses reliability data (failure probability) in a qualitative natural language and mathematically represented by a membership function of fuzzy numbers. That way, a defuzzification technique is needed to convert the membership function into a reliability score, and later into their corresponding fuzzy failure rates, which is a good approximation for probabilistic failure rates [36]. We use an area defuzzification technique (ADT), since it presented the best results in Reference [36].

Let $f_{f}=\left(a,b,c,d\right)$ be a fuzzy function, where a,b,c,d are the parameters of a trapezoidal membership function. The parameter $f_{f}$ can be converted to a crisp score by ADT (Equation (11)) and this score can be used to estimate the link failure rate associated to $f_{f}$ according to Equation (12). We use the PRR available in Reference [9] to define the values of the membership function of each link based on the fact that the higher is the PRR, the lower is the probability of the link to fail.

It is important to remark that, according to Purba et al. [36], the relation between the qualitative natural language of the failure probability and its mathematical representation through the membership function (defined by fuzzy function parameters *a, b, c, d*) must be stated by experts’ belief of the most expected value that a failure event may occur. That way, the association between these parameters was a relatively subjective choice of us, the authors, based on our familiarity, considerable training in and knowledge of this application field. The same was applied to the association between these parameters and the PRR.

Thereby, we consider that a PRR under 50% indicates a down link (nonoperational link). The fuzzy function was proportionally generated over the range of 50–100% and the link failure rates ($\lambda_{lk}$) were computed through Equations (11) and (12), according to Purba et al. [36]. These rates, as well as the other parameters of the fuzzy model approach, are shown in Table 2. Once a communication failure occurs, due to the transient nature of links, the connection from a node to the gateway node could be reestablished naturally, eventually, or by the re-routing or self-healing features of routing protocols, which lead us to consider in this scenario the link repair rate $\mu_{lk}$ = 4, which means about 15 min to reestablish.

(11) $$\begin{array}{cc} & ADT={\left[18{\left(a+b-c-d\right)}^{2}\right]}^{-1}\times [\left(a+2b-2c-d\right)\times \\ \; & \times \left({\left(2a+2b\right)}^{2}+\left(c+d\right)\left(-3a+2c-d\right)-2c\left(3b+d\right)-4ab\right)] \end{array}$$

(12) λlk=10−1−ADT1−ADTADTADT11/33×2.301,ADT≠00,ADT=0.

### 5.2. Dependability Evaluation

**Figure 11:**
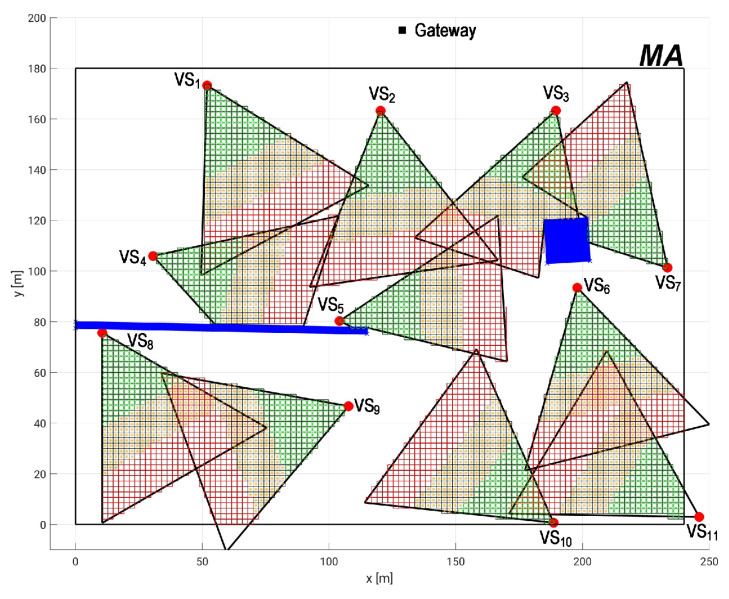
Network deployment considering QoM under visual failures.

Once the dependability parameters are set, the application requirements need to be defined to assess the system dependability. In this case, it is necessary to define the minimum acceptable area coverage ($CA_{mb}$) and quality of monitoring (AQM), $CA_{min}$ and $AQM_{min}$, respectively. For this, based on the layout shown in Figure 9, we consider two obstacles on the monitoring area: a wall separating a management station to the chemical processes and a forklift that spends most part of time near to a conveyor belt to get the paperboard boxes. Figure 11 shows the network deployment, including the obstacles (blue rectangles) and the quality perspective of MA. Notice that the FoV of visual sensors $vs_{3}$ and $vs_{7}$ are partially occluded by the forklift and the FoV of visual sensors $vs_{4}$ and $vs_{5}$ are partially occluded by the wall. The coverage area is $CA_{mb}=21,691$ m${}^{2}$, which is approximately 50% of total MA, and the quality of monitoring is approximately AQM=28%. Since in this work it is not considered network redeployment, the maximum coverage area and the maximum quality of monitoring are those ones presented by the network deployment. This means that $CA_{min}\leq 50\%$ and $AQM_{min}\leq 28\%$, otherwise this network will not fulfill the application requirements.

**Figure 12:**
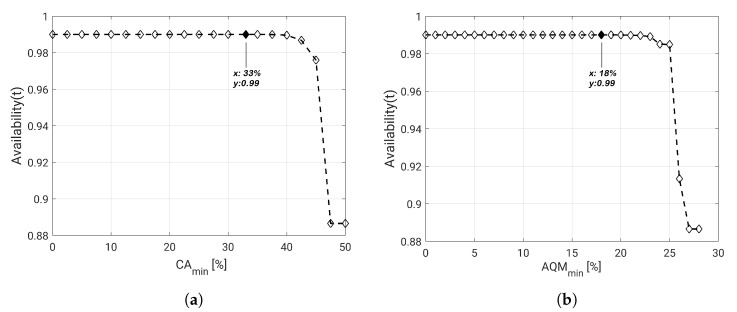
Impact of (**a**) *CA_min_* and (**b**) *AQM_min_* on dependability evaluation.

In order to evaluate the impact of visual failures on dependability, first we perform the system availability assessment considering that $AQM_{min}=0\%$, varying $CA_{min}$ from 0% to the maximum value of the area able to be covered by the network deployment (50%). In this case, we intend to verify how large is the portion of area of the monitored field that the system can cover considering the proposed deployment. We can observe in Figure 12a that the system presents an acceptable availability (>99%) for values of $CA_{min}$ from 0% to 33%.

Then, the same analysis is performed considering $CA_{min}=0\%$ and varying $AQM_{min}$ from 0% to the maximum value of quality of the monitoring provided by the network deployment ( 28%). Here we intend to verify how well the monitoring field is covered considering the proposed deployment. We can observe in Figure 12b that the system presents an acceptable availability (>99%) for values of $AQM_{min}$ from 0% to 28%. This analysis implies that, if an application intends to implement the proposed deployment, it will have a considerably satisfactory availability if its requirements of coverage area and monitoring area are $CA_{min}\leq 33\%$ and $AQM_{min}\leq 18\%$, respectively.

Actually, dependability is assessed here in terms of availability. Notice that, in order to perform a system reliability evaluation, it is only necessary to disregard the repair activities, which means setting the repair rates $\mu_{hw}$, $\mu_{bt}$ and $\mu_{lk}$ equal to zero.

**Figure 13:**
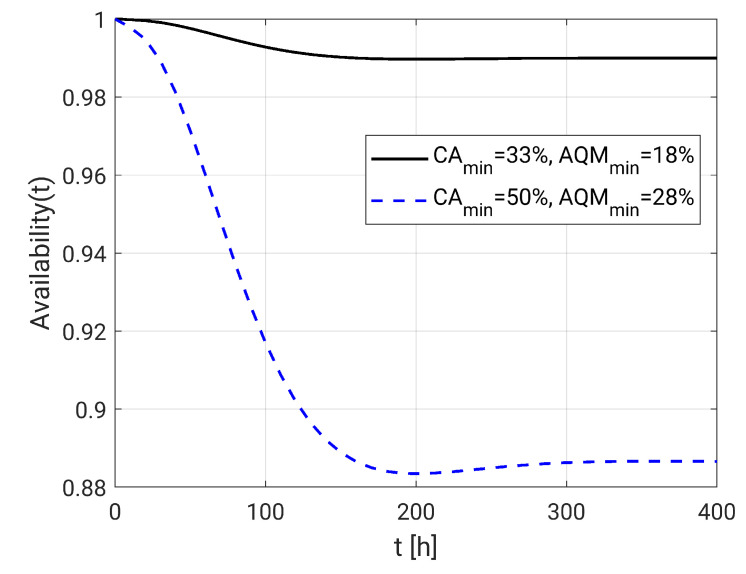
Availability at minimum and maximum acceptable visual failures.

Next, we evaluate the system availability with the maximum values of $CA_{min}$ and $AQM_{min}$ that guarantee 99% of availability, which are $CA_{min}=33\%$ and $AQM_{min}=18\%$, and the maximum possible values, which are the coverage values of network deployment ($CA_{min}=50\%$ and $AQM_{min}=28\%$). With this, it is possible to verify, respectively, the maximum and minimum availability response which the system can present for the considered deployment. The Figure 13 shows the respective steady availability values, 88.66866% and 99.00779%.

If we consider that the nodes are supplied by a power line instead of a battery, we can re-evaluate the system dependability without the effect of batteries. This can be achieved by removing the battery Markov Chain from the model or simply by setting the battery discharge rates ($\lambda_{bt}$) to zero, avoiding to change the fault tree model. In this case, the system availability increases to 99.97261% and 99.67175%, respectively, achieving expected results of a well formed WirelessHART network [40].

**Figure 14:**
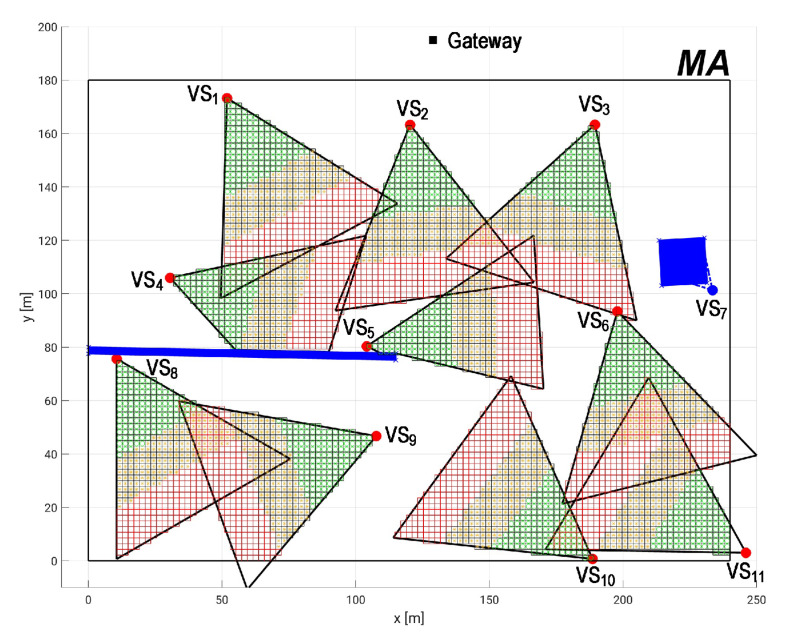
Network deployment with considerable FoV occlusion.

Finally, it is analyzed the impact of transforming sensors with low coverage ($OFoV_{vs}<FoV_{min}$) into relay sensors. Figure 14 shows an example where the sensor $vs_{7}$ is occluded and its OFoV is less than 5%. In this case we consider that this sensor cannot provide enough visual information and then its camera is turned off to save battery, but the node keeps working re-transmitting messages. We analyze the worst case scenario, that is, the maximum possible values of $CA_{min}$ and $AQM_{min}$, which is the coverage values of network deployment ($CA_{min}=47\%$ and $AQM_{min}=26\%$). In this case, the application availability considering $vs_{7}$ as a visual sensor node is 89.56148%. When the sensor is considered as a relay node, the assessed availability is 89.56149%. This is a very small increase in terms of the absolute values, although, in terms of dependability, it is very significant, which shows that the proper management of power consumption turning some visual nodes into relay nodes compensates somehow the visual failure.

## 6. Conclusions

In this article, we proposed a comprehensive methodology for dependability evaluation in wireless sensor networks considering hardware, networking and visual coverage failures. While hardware and network failures had been already considered in other works, visual failures were addressed in this article as an important element of dependability, since they are related to the inability of the sensors to perform sufficient area coverage respecting the minimum required quality of monitoring. Moreover, in order to make this problem computationally tractable for large networks, an efficient algorithm for area coverage and QoM computation was proposed, bringing then an important contribution to this area.

As additional contributions, we also addressed an important issue, which is practical aspects of parameters setting. Moreover, it was considered the study case of a paper mill with a WirelessHART network. The methodology provided coherent results for an industrial context, pointing out important development directions in future works.

We believe that industrial WSN applications can significantly benefit from the proposed approach. In fact, we expect not only a more efficient planning of new monitoring applications in these scenarios, but also a continued understanding of how failures may impact the final quality and how and where improvements can be performed.

As future works, an interesting research area would be to analyze the systems’ dependability in runtime, during an application operation. Doing so, we could dynamically analyze a system’s dependability, tracking system alterations due to failures or repairs, as soon as they happen. This dynamic evaluation context also allows the inclusion of mobile obstacles in the mathematical model, making the methodology useful for more realistic applications. Also, the system dependability could be improved by an optimization process with some nodes parameters being altered (cameras’ orientation, nodes’ position, link quality, power consumption), aiming at dependability improvement in case of a bad deployment configuration or in response to changes in obstacles positions.

## Figures and Tables

**Table 1:** Visual coverage research in dependability of wireless sensor networks.

Work	QoM	Occlusion	WSN Evaluation	VCF	Brief Description
He et al. [12] and Hsiao et al. [13]	X	–	–	–	Area coverage analysis aiming at full-view coverage, considering QoM by the facing angle of points of interest.
Konda et al. [14]	X	–	–	–	Automatic deployment of WVSN to maximize coverage and visual quality in indoor environments, considering perspective distortion of the acquired images.
Shriwastav and Song [15]	X	–	–	–	UAV network for area coverage. Network parameters are carefully handled during redeployment to keep the coverage quality constant.
Tao et al. [6]	X	X	–	–	QoM-enhancing coverage scheme in a full area coverage scenario, considering the different area importance and the weighted quality of captured image.
Jesus et al. [5]	X	X	–	–	Proposal of metrics to QoM assement and optimization in WVSN for area coverage. The QoM model is realistic and flexible to guide (re)deployment.
Scott et al. [16]	X	X	–	–	Occlusion-aware area coverage with aerial sensing quality, providing satisfactory spatial resolution subject to the energy constraints of UAVs.
Costa et al. [17]	–	X	–	X	Selection of redundant visual nodes for enhanced resistance to visual failures, considering occlusion caused by obstacles modeled by lines.
Jesus et al. [11]	–	X	–	X	Selection of faultless visual nodes based on the amount of monitored area, considering occlusion caused by mobile obstacles modeled by rectangles.
Andrade and Nogueira [18]	–	–	X	–	Petri net-based approach for modeling and analysis of dependability on IoT networks for disaster recovery.
Silva et al. [19]	–	–	X	–	Integrative methodology for dependability evaluation of industrial WSN, based on fault tree analysis, considering hardware and communication failures.
Costa et al. [20]	–	–	X	X	Integrative methodology for dependability evaluation of WVSN, based on fault tree analysis, considering hardware, communication and visual failures.
Jesus et al. [10]	–	–	X	X	Automated integrative methodology for dependability evaluation of WVSN, based on fault tree analysis, considering hardware, communication and visual failures.
Proposed approach	X	X	X	X	Automated integrative methodology for dependability evaluation of WVSN, based on fault tree analysis, considering hardware, communication and visual failures, related to occlusion and QoM.

**Table 2:** Parameters of link reliability and fuzzy model approach.

PRR	Failure Probability	Fuzzy Function Parameters (a,b,c,d)	$\lambda_{\mathrm{lk}}$
0–50%	Down link	(0, 0, 0, 0)	*∞*
50–60%	Very High	(0.94, 0.95, 1, 1)	$4.3962\times 10^{4}$
60–70%	High	(0.865, 0.875, 0.925, 0.935)	$6.2411\times 10^{4}$
70–80%	Reasonably High	(0.77, 0.80, 0.85, 0.86)	$9.4357\times 10^{4}$
80–90%	Moderate	(0.715, 0.725, 0.775, 0.785)	$1.3609\times 10^{3}$
90–95%	Reasonably Low	(0.64, 0.65, 0.70, 0.71)	$1.5641\times 10^{3}$
95–98%	Low	(0.565, 0.575, 0.625, 0.635)	$2.5627\times 10^{3}$
98–100%	Very Low	(0.5, 0.5, 0.55, 0.56)	$3.6855\times 10^{3}$
